# Kinome-Wide siRNA Screening Identifies Src-Enhanced Resistance of Chemotherapeutic Drugs in Triple-Negative Breast Cancer Cells

**DOI:** 10.3389/fphar.2018.01285

**Published:** 2018-11-09

**Authors:** Yen-Dun Tony Tzeng, Pei-Feng Liu, Ju-Yueh Li, Li-Feng Liu, Soong-Yu Kuo, Chiao-Wei Hsieh, Cheng-Hsin Lee, Chih-Hsuan Wu, Michael Hsiao, Hong-Tai Chang, Chih-Wen Shu

**Affiliations:** ^1^Department of Surgery, Kaohsiung Veterans General Hospital, Kaohsiung, Taiwan; ^2^Institute of Clinical Medicine, National Yang-Ming University, Taipei, Taiwan; ^3^Department of Medical Education and Research, Kaohsiung Veterans General Hospital, Kaohsiung, Taiwan; ^4^Department of Nursing, Shu-Zen Junior College of Medicine and Management, Kaohsiung, Taiwan; ^5^Department of Obstetrics and Gynecology, Kaohsiung Veterans General Hospital, Kaohsiung, Taiwan; ^6^School of Medicine for International Students, I-Shou University, Kaohsiung, Taiwan; ^7^Institute of Biological Science & Technology, I-Shou University, Kaohsiung, Taiwan; ^8^Department of Biotechnology, Fooyin University, Kaohsiung, Taiwan; ^9^Genomics Research Center, Academia Sinica, Taipei, Taiwan; ^10^Department of Biochemistry, College of Medicine, Kaohsiung Medical University, Kaohsiung, Taiwan; ^11^Institute of Biomedical Sciences, National Sun Yat-sen University, Kaohsiung, Taiwan

**Keywords:** kinome, siRNA, high-throughput screening, Src kinase, chemoresistance, triple-negative breast cancer

## Abstract

**Background:** Chemotherapy is the main treatment for triple-negative breast cancer (TNBC), which lack molecular markers for diagnosis and therapy. Cancer cells activate chemoresistant pathways and lead to therapeutic failure for patients with TNBC. Several kinases have been identified as chemoresistant genes. However, the involvement of kinases in the chemoresistance in TNBC cells is not fully understood.

**Methods:** We employed a kinome siRNA library to screen whether targeting any kinases could increase the chemosensitivity of TNBC cell lines. The effects of kinase on cell viability in various breast cancer cells were validated with ATP level and colony formation. Protein expression and phosphorylation were determined by immunoblotting. The Cancer Genome Atlas (TCGA) dataset was collected to analyze the correlation of Src expression with prognosis of TNBC patients.

**Results:** Primary screening and validation for the initial hits showed that Src kinase was a potential doxorubicin-resistant kinase in the TNBC cell lines MDA-MB-231 and Hs578T. Both siRNA against Src and the Src inhibitor dasatinib enhanced the cytotoxic effects of doxorubicin in TNBC cells. Moreover, phosphorylation of AKT and signal transducer and activator of transcription 3 (STAT3), downstream effectors of Src, were accordingly decreased in Src-silenced or -inhibited TNBC cells. Additionally, TCGA data analysis indicated that Src expression levels in tumor tissues were higher than those in tumor-adjacent normal tissues in patients with TNBC. High co-expression level of Src and STAT3 was also significantly correlated with poor prognosis in patients.

**Conclusion:** Our results showed that Src-STAT3 axis might be involved in chemoresistance of TNBC cells.

## Introduction

Breast cancer is a heterogeneous disease with an increasing incidence worldwide (Bianchini et al., 2016). Three markers aberrantly expressed in breast cancer are used for targeted therapy in clinics, including the hormone receptors for estrogen (ER) and progesterone (PgR) and the human epidermal growth factor receptor 2 (Her2). Triple-negative breast cancer (TNBC), characterized by tumors lacking ER, PgR, and Her2 expression, represents approximately 15% of all breast cancer cases (Collignon et al., 2016). TNBC belongs to basal-like subtype of breast cancer and is associated with higher genetic instability, which does not have molecules for targeted therapy so far (Alcala-Corona et al., 2017). Chemotherapy is the major treatment for patients with TNBC (Bianchini et al., 2016). Though standard anthracycline and taxane-based chemotherapy can achieve a pathological complete response (pCR) for 30–40% of patients with early stage TNBC (von Minckwitz et al., 2012), many patients with TNBC do not respond to chemotherapy and have relatively poor outcomes and prognosis (Bonotto et al., 2014). Moreover, several groups currently investigate chemoresistant pathways in TNBC cells, suggesting chemoresistance is still a challenging problem in more advanced patients with TNBC (Loi et al., 2013; Wein and Loi, 2017).

Protein kinases play a crucial role in signal transduction for cell proliferation, survival and migration, which are common features of cancer cells. Aberrant expression or activation of kinases may promote tumorigenesis, malignancy and drug resistance. Several oncogenic kinases have been discovered in different types of cancer. For example, BCR-ABL plays a role in chronic myeloid leukemia, epidermal growth factor receptor (EGFR) in lung cancer, HER2 in breast cancer and the B-Raf mutation in melanoma (Ascierto et al., 2012). Highly selective inhibitors for these kinases have also been developed for cancer treatment as targeted therapies (Knight et al., 2010). Of note, there are approximately 538 kinase genes in the human genome (Cheng et al., 2014). Only a few of the kinases have been well studied in various types of cancer. As such, little is known about the roles of most of the kinases in cancer malignancy and drug resistance, particularly in TNBC cells.

Gene silencing with small interfering RNAs (siRNAs) provides a powerful tool to identify novel regulators required for many biological processes, such as cell survival and death (Gao et al., 2014). To examine if any kinases represent potential chemoresistance genes in TNBC cells (Foulkes et al., 2010), we used MDA-MB-231 cells expressing luciferase to monitor cell viability in live cells, and screened MDA-MB-231 with a kinome siRNA library to identify potential drug resistance kinases in TNBC cells treated with chemotherapy. Genetic or pharmacological ablation of the Src kinase diminished the phosphorylation of downstream effectors, AKT and STAT3, and enhanced the sensitivity of TNBC cells to chemotherapeutic drugs. Co-expression of high levels of Src and STAT3 was associated with poor prognosis in patients with TNBC, suggesting that combination therapy with Src inhibitor and chemotherapeutic drugs might be beneficial for Src-expressing patients with TNBC.

## Materials and Methods

### Cell Culture and Transfection, and Stable Selection

MDA-MB-231 and Hs578T breast cancer cells were cultured in DMEM with 10% FBS, 100 μg/ml streptomycin, 100 IU penicillin, and 1% L-glutamine at 37°C in 5% CO_2_ and 95% air. For gene knockdown with siRNA, cells were transfected with 5 nM scramble siRNA or siRNA against Src kinase (6714, Dharmacon) for 72 h using lipofectamine RNAiMAX (Invitrogen, 13778-150). For lentiviral infection, HEK293T cells were seeded into 6-well plates then transfected with 2 μg pCDH-EF1-MCS-T2A-Puro (CD520A1, System Bioscience) encoding a luciferase expression vector, scramble shRNA or shRNA against Src (TRCN0000195339) using 6 μl lipofectamine 2000 (Invitrogen, 11668027) for 16 h. The transfected cells were then rinsed with media and incubated for 48 h. The supernatant was further used to infect MDA-MB-231 cells, which were maintained in culture with 1 μg puromycin added every 2–3 days to obtain stable cell lines.

### Cell Viability Assays

For live cell viability assays with siRNA screening, MDA-MB-231 cells harboring a luciferase plasmid (2000 cells/40 μl) were seeded into each well of 384-well white plates containing 10 nM scramble siRNA or kinome siRNA library (2127 siRNA for 709 gene, A30079, Thermo Fisher Scientific) and RNAiMAX (Invitrogen, 13778-150) for 48 h. The cells were mixed with 200 μM D-luciferin (E1605, Promega) and treated with or without doxorubicin for 24 h. The net luminescent signal (RLU) between 48 and 72 h was monitored to reflect cell viability (as measured by the ATP level). For cells without a luciferase reporter plasmid, the cells were treated with dasatinib (S1021, Selleckchem) in the presence or absence of doxorubicin (324380, Calbiochem) for 24 h and mixed with Cell-Titer Glo (G7572, Promega) for 10 min. The luminescent signal was read with a Fluoroskan Ascent FL reader (Thermo Fisher Scientific).

### Tumorsphere Formation and Live/Dead Assay

MDA-MB-231 and Hs578T cells were seeded at density of 2 × 10^4^ in NanoCulture plates (SCIVAX Corporation, Kanagawa, Japan) in the presence of 10 nM siRNA. The cells were cultured for 3 days until tumorsphere formation. The tumorsphere were then treated with anticancer drugs for 48 h and stained with Calcein AM (Green) and Ethidium homodimer-1 (EthD-1, Red) (LIVE/DEAD Viability/Cytotoxicity Kit, Thermo Fisher Scientific) for live and dead cell population, respectively. The cells treated with or without 70% methanol for 30 min were considered as a relative control for all (100%) dead or live cells. The live and dead cells were then observed under microscopy and quantitated with Fluoroskan Ascent FL reader (Thermo Fisher Scientific) as previously described (Liu et al., 2018).

### Real-Time PCR

The cells were transfected with 10 nM scramble siRNA or siRNA against kinases for 48 h. The cells were then harvested to extract total RNA with TRIzol Reagent (Invitrogen, 15596-018). The RNA was then reverse-transcribed with SuperScript II RNase H-Reverse Transcriptase (Invitrogen, 18064-014). The mRNA level of each gene was amplified with specific primers and SYBR Green Master Mix (Applied Biosystems, 4385612). The results were analyzed by a StepOnePlus^TM^ system (Applied Biosystems) using GAPDH as normalization control. The primer sequences will be provided upon request.

### Western Blot for Protein Expression

The cells were briefly rinsed in PBS and lysed with RIPA buffer (1% NP40, 50 mM Tris Cl pH 7.5, 150 mM NaCl, 0.25% sodium deoxycholate 1% SDS, and a protease inhibitor cocktail). The cell lysates were resolved by SDS-PAGE and transferred electrophoretically onto nitrocellulose membranes. The membranes were blocked with bovine serum albumin (BSA) for the anti-phosphoserine antibody and with 0.1% casein in 0.2X PBS for the other antibodies. The membranes were then incubated with the primary antibodies, including anti-Src (2109, cell signaling), anti-pSrc (6943, Cell Signaling), phospho-Stat-3 (9145, cell signaling), and anti-ACTB (A5441, Sigma) at 4°C overnight. The proteins were probed with HRP-labeled secondary antibody and detected with an ECL reagent. The membrane was scanned and analyzed for protein expression with a BioSpectrum^®^ Imaging System (UVP, Upland, CA, United States).

### Clonogenic Assays

Breast cancer cells were seeded in 12-well plates at a density of 5 × 10^3^ cells/well in the presence of scramble siRNA or siRNA. The culture media was renewed every 3 days until colony formation. The cell colonies were fixed with 2% paraformaldehyde, stained with crystal violet (0.25% w/v) and counted to determine the cytotoxic effects of cells.

### Statistics

The data are reported as the mean ± SEM from at least three independent experiments. To compare cell viability between control and Src-silenced cells, non-parametric 2-tailed Student *t*-test is used to evaluate significance. To analyze gene expression in TNBC patients’ dataset obtained from the UCSC Xena website^1^, Wilcoxon signed-rank test was used to evaluate the different levels of Src kinase between the tumors and corresponding tumor adjacent normal tissues. To ensure that each group had enough cases in the survival analyses, the ROC and 25th percentile cutoffs were set for Src and STAT3, respectively. Cumulative survival curves were estimated using the Kaplan–Meier method. Univariate and multivariate Cox proportional hazards models were used for crude and adjusted hazard ratios, respectively. A *p* < 0.05 (2-sided) was considered significant.

## Results

### High-Throughput Screening for Potential Kinases Involved in Chemoresistance of TNBC Cells

**FIGURE 1:**
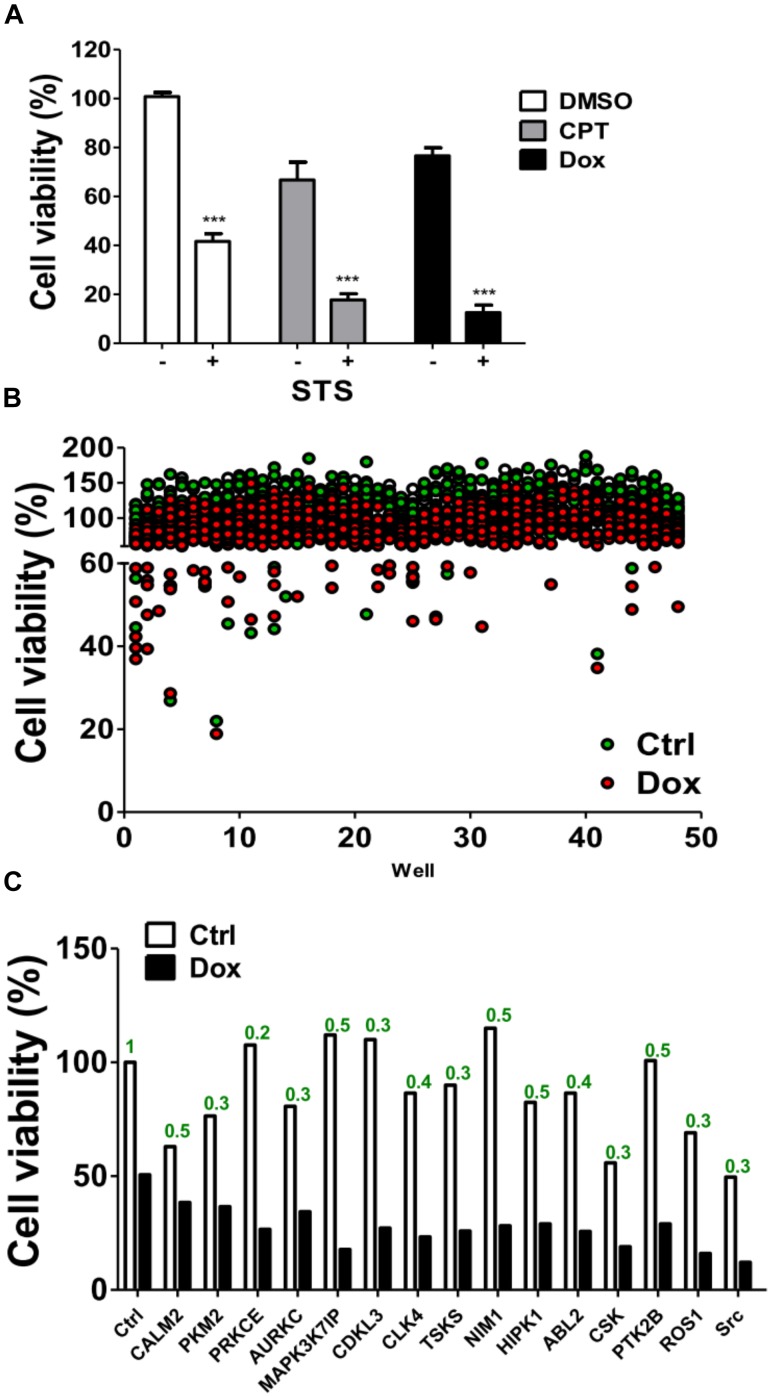
Screening of a kinome siRNA library for kinases involved in chemoresistance in TNBC cells. **(A)** The triple-negative breast cancer cell line MDA-MB-231 harboring a luciferase expression vector was treated with chemotherapeutic drugs including camptothecin (CPT, 10 μM) and doxorubicin (Dox, 1 μM) in the presence or absence of 20 nM STS for 48 h. The luciferase was measured with cell permeable D-luciferin to monitor cell viability in live cells. **(B)** MDA-MB-231 cells were seeded into 384-well plates containing pooled siRNA (10 nM) against each single kinase gene for 48 h and treated with (red dots) or without (green dots) doxorubicin (1 μM) for 24 h. The cell viability of treated cells was measured by Cell-Titer Glo. **(C)** The top 15 hits that were resistant to doxorubicin in MDA-MB-231 cells are shown. The mRNA levels for each gene as labeled were determined by real-time PCR and analyzed by Prism 5.0 software using scramble siRNA as a control. ^∗∗∗^represents *p* < 0.001.

**FIGURE 2:**
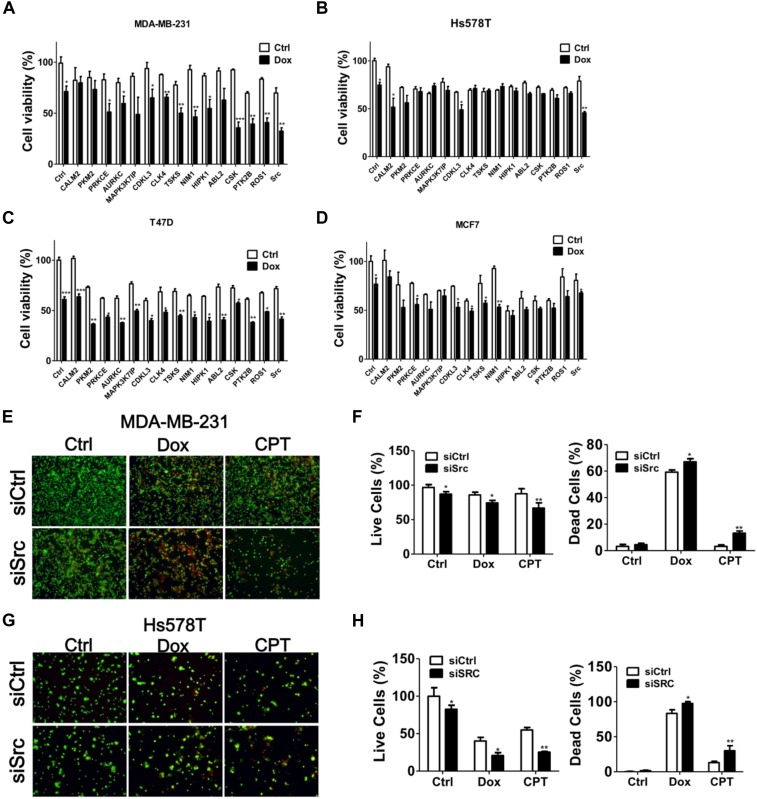
Hit validation with several breast cancer cell lines. TNBC cell lines **(A)** MDA-MB-231 and **(B)** Hs578T or **(C)** T47D, and **(D)** MCF7 were transfected with siRNA against the kinase hits identified above for 48 h and treated with doxorubicin (1 μM) for 24 h. The cytotoxic effects of the combination of doxorubicin and gene silencing in treated cells were determined with Cell-Titer Glo. In contrast to non-treated cells (Ctrl), the significant effects of siRNA against top 15 genes in cells were shown as ^∗^*p* < 0.05, ^∗∗^*p* < 0.01 or ^∗∗∗^*p* < 0.001. **(E)** MDA-MB-231 and **(F)** Hs578T cells were cultured in nanoparticle plates in the presence of scramble siRNA or siSrc for 3 days to form tumorspheres. The tumorspheres were further treated with doxorubicin (1 μM) or camptothecin (CPT, 10 μM) and death effects were determined by Live/Dead cytotoxicity assay. The representative results were shown and quantified in panel (**G**, MDA-MB-231) and (**H**, Hs578T) for live (Green) and dead (Red) cell populations. The live and dead cell populations were counted separately and calculated as percentage using untreated and methanol-treated cells as all (100%) live and dead cells, respectively. The data are expressed as the mean ± SEM from three independent experiments. In contrast to tumorspheres with scramble siRNA (siCtrl), the significant effects of chemosensitivity in tumorspheres with siRNA against Src were shown as ^∗^*p* < 0.05, ^∗∗^*p* < 0.01.

MDA-MB-231 is a TNBC cell line and is reportedly resistant to many anti-cancer drugs (Totary-Jain et al., 2012; Yu et al., 2013). To evaluate if any kinases are involved in the chemoresistance of TNBC cells, MDA-MB-231 cells were treated with chemotherapeutic drugs camptothecin and doxorubicin in the presence or absence of staurosporine (STS; Figure 1A), a pan kinase inhibitor that wildly suppress many kinases at low dose (Meggio et al., 1995). STS reduced the cell viability and synergized the cytotoxic effects of the chemotherapeutic drugs, particularly of doxorubicin (Figure 1A), suggesting that kinases may play a role in the chemoresistance of MDA-MB-231 cells. To further examine which kinase was involved in the survival of doxorubicin-treated MDA-MB-231 cells, MDA-MB-231 cells expressing luciferase were used to monitor cell viability in live cells and to screen a kinome siRNA library in the presence or absence of doxorubicin (Figure 1B). We selected 15 top-ranked genes due to knockdown of these genes enhanced cytotoxicity of doxorubicin in MDA-MB-231 cells, which were likely responsible for the resistant effects of doxorubicin in TNBC cells (Figure 1C). The knockdown efficiency of these genes was confirmed by real-time PCR (Figure 1C). The 15 genes were further confirmed with parental TNBC cells (MDA-MB-231 and Hs578T), and their cell viability was measured with Cell-Titer Glo (Figures 2A,B). Although knockdown of several genes significantly enhanced doxorubicin-induced cytotoxicity in MDA-MB-231 cells, only silencing Src kinase augmented doxorubicin-induced cytotoxicity in both MDA-MB-231 and Hs578T cells (Figures 2A,B). We also examined the kinome hits in non-TNBC breast cancer cells, which included T47D and MCF-7 cells (Figures 2C,D). The genes involved in chemoresistance varied in different breast cancer cell lines. Nevertheless, knockdown of Src kinase enhanced the cytotoxicity of doxorubicin in both T47D and MCF-7 cells (Figures 2C,D). Furthermore, we knockdowned Src to determine its chemoresistant effects in TNBC cells with tumorsphere culture model, which mimics *in vivo* condition and a feature of cancer stem cells (Shaheen et al., 2016). Silencing Src decreased the live cells population, while it increased the dead cells populations in TNBC cells when exposed to chemotherapeutic drugs, including camptothecin and doxorubicin (Figures 2E–H).

### Src Deprivation Increased Chemosensitivity in TNBC Cells

**FIGURE 3:**
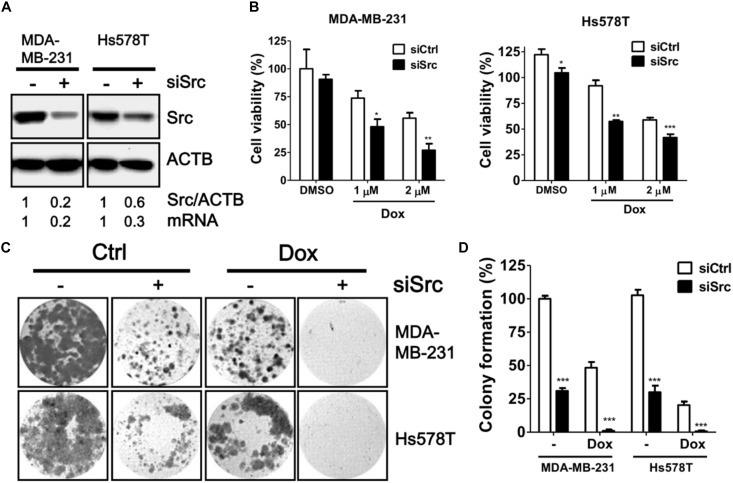
Effects of siRNA against Src in doxorubicin-treated TNBC cells. **(A)** Triple-negative breast cancer cell lines MDA-MB-231 and Hs578T were transfected with siRNA against Src kinase for 48 h. The knockdown efficiency of Src in the cells was examined with immunoblotting and real-time PCR. **(B)** The Src-silenced MDA-MB-231 and Hs578T cells were exposed to doxorubicin (1 or 2 μM) for 24 h to evaluate the cytotoxicity with Cell-Titer Glo. **(C)** The Src-silenced MDA-MB-231 and Hs578T cells were exposed to doxorubicin for 1 h and allowed to recover for 14 days. The cells were fixed and stained with crystal violet to count the number of colonies formed. **(D)** The quantitative results are expressed as the mean ± SEM from three independent experiments. ^∗^*p* < 0.05, ^∗∗^*p* < 0.01, ^∗∗∗^*p* < 0.001 vs. non-targeting control siRNA (siCtrl).

**FIGURE 4:**
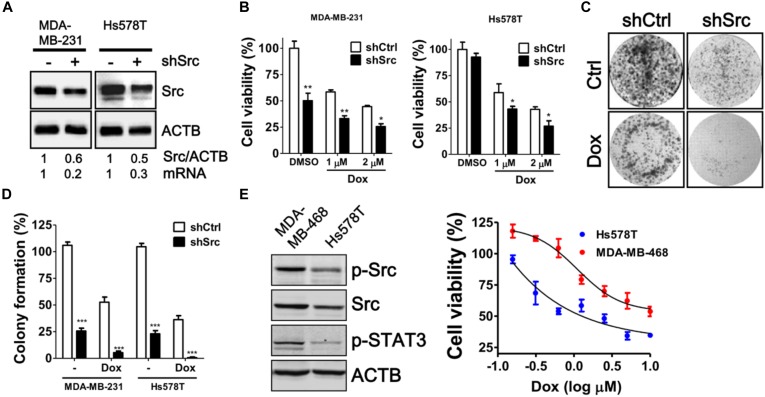
Cytotoxic Effects of doxorubicin in stable Src-silenced TNBC cells. **(A)** MDA-MB-231 and Hs578T cells were infected with shRNA against Src kinase, and a single stable clone for each cell line was selected with puromycin. The knockdown efficiency of Src in the cells was examined with immunoblotting and real-time PCR. **(B)** The Src-silenced MDA-MB-231 and Hs578T cells were exposed to doxorubicin (1 or 2 μM) for 24 h to evaluate the cytotoxicity with Cell-Titer Glo. **(C)** The Src-silenced MDA-MB-231 cells were exposed to doxorubicin (1 μM) for 1 h and allowed to recover for 14 days. The cells were fixed and stained with crystal violet to count the number of colonies formed. **(D)** The quantitative results are expressed as the mean ± SEM from three independent experiments. ^∗^*p* < 0.05, ^∗∗^*p* < 0.01, ^∗∗∗^*p* < 0.001 vs. non-targeting control shRNA (shCtrl). **(E)** Src expression in MDA-MB-468 and Hs578T was examined with immunoblotting and dose-responsive curves of doxorubicin for TNBC cells were shown.

To verify the knockdown efficiency of Src kinase as mRNA level at the protein level, the protein level of Src kinase was determined in MDA-MB-231 and Hs578T cells transfected with siRNA against Src kinase by immunoblotting (Figure 3A). Src-silenced cells had significantly increased sensitivity to doxorubicin in both MDA-MB-231 and Hs578T cells in a dose-dependent manner (Figure 3B). Moreover, clonogenic assay results showed that the number of colonies was decreased in doxorubicin-treated, Src-silenced cells. A combination of siRNA against Src and doxorubicin treatment completely abrogated colony formation in MDA-MB-231 and Hs578T cells (Figures 3C,D). To avoid side effects of transient transfection, we generated Src stable knockdown cells with shRNA against Src in MDA-MB-231 and Hs578T cells (Figure 4). Similar to the effects of siRNA on cell viability and colony forming ability, stably Src-silenced TNBC cells were more sensitive to doxorubicin treatments than control cells (Figures 4B–D). Further, MDA-MB-468, another Src highly expressed and activated TNBC cell line, was even more resistant to doxorubicin (Figure 4E), suggesting Src might be associated with chemoresistance in TNBC cells.

### Src-STAT3 Axis in Chemoresistance in TNBC Cells

**FIGURE 5:**
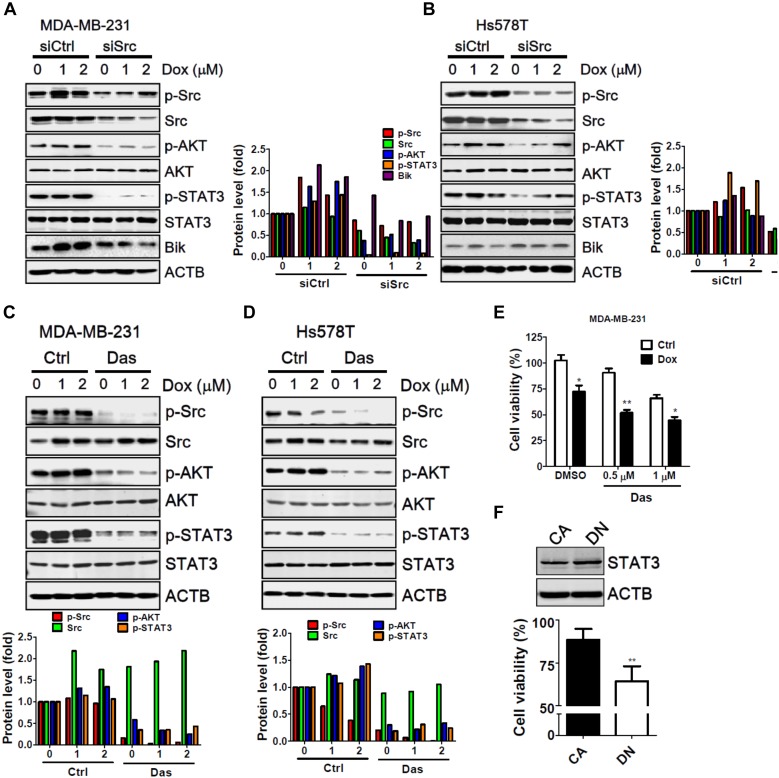
The involvement of AKT and STAT3 phosphorylation in Src-silenced TNBC cells. **(A)** MDA-MB-231 and **(B)** Hs578T TNBC cells were transfected with siRNA against Src kinase for 72 h and then exposed to doxorubicin (1 or 2 μM) for 2 h. The cells were harvested to examine the activation or induction of potential downstream effectors of Src, including p-AKT, p-STAT3, and Bik, with immunoblotting. Actin was used as a normalization control. **(C)** MDA-MB-231 and **(D)** Hs578T TNBC cells were treated with doxorubicin (1 or 2 μM) for 2 h in the presence or absence of the Src inhibitor dasatinib (Das, 1 μM). The cells were harvested to examine the phosphorylation of Src, AKT, and STAT3 with immunoblotting. Actin was used as a normalization control. The quantitative results are expressed as the mean from three independent experiments. **(E)** MDA-MB-231 TNBC cells were treated with doxorubicin (1 μM) for 24 h in the presence or absence of the Src inhibitor dasatinib (Das, 0.5 or 1 μM). The cytotoxic effects of treated cells were determined with Cell-Titer Glo. **(F)** MDA-MB-231 cells were transfected with vector encoding STAT3 constitutive active (CA) or dominant negative mutant (DN) for cytotoxic effects of doxorubicin with Cell-Titer Glo. The results are expressed as the mean ± SEM from three independent experiments. ^∗^*p* < 0.05, ^∗∗^*p* < 0.01 vs. control cells (Ctrl).

Src was the first discovered oncogenic tyrosine kinase due to its ability to promote tumor initiation and malignancy. The Src kinase can lead to drug resistance in cancer cells during targeted therapy through the activation of several downstream effectors, particularly of AKT and STAT3 (Zhang and Yu, 2012). In addition, recent studies indicate that Src triggers the ubiquitination and degradation of BH3 only protein Bik through the Ras-Raf-ERK pathway to attenuate apoptosis in cells under stress (Lopez et al., 2012; Ballesta et al., 2013). To identify any potential molecules involved in the chemoresistance of Src, MDA-MB-231 and Hs578T cells were transfected with siRNA against Src and treated with doxorubicin (Figure 5). Silencing of Src kinase largely decreased the protein level of total and phosphorylated Src in MDA-MB-231 cells. Additionally, phosphorylation of AKT and STAT-3 was accordingly diminished in cells with and without treatment of doxorubicin (Figure 5A). Likewise, similar results were seen for AKT and STAT3 at the protein level in Src-silenced Hs578T cells (Figure 5B). Nevertheless, Bik protein levels were unexpectedly decreased in Src knockdown MDA-MB-231 cells, while it was slightly increased in Hs578T cells (Figure 5). In addition, dasatinib, a selective Src family kinase inhibitor, attenuated the phosphorylation of AKT and STAT3 in MDA-MB-231 and Hs578T cells (Figures 5C,D). Dasatinib further inhibited cell viability and enhanced doxorubicin sensitivity in MDA-MB-231 cells (Figure 5E). Ectopically expression of STAT3 constitutive active form (CA) was more resistant to doxorubicin than dominant negative form (DN) in MDA-MB-231 cells (Figure 5F), suggesting that Src-STAT3 signaling may confer to resistance of doxorubicin in TNBC cells.

### The Association of Src and STAT3 Expression With Survival in Patients With TNBC

**FIGURE 6:**
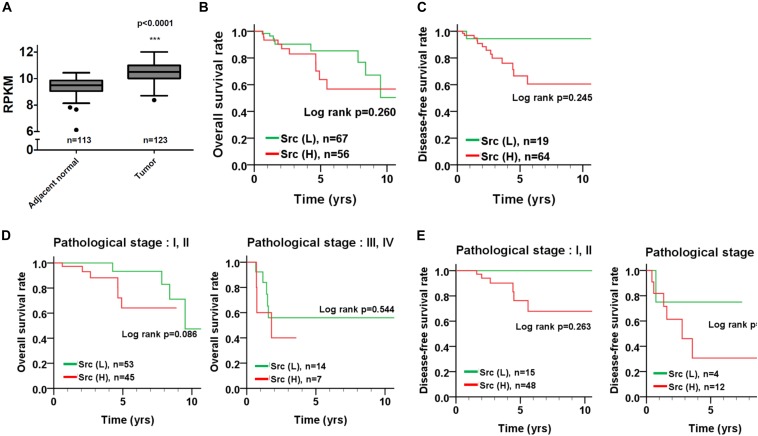
Src gene expression and its association with survival curves in patients with TNBC. **(A)** Src gene expression was analyzed from TCGA TNBC dataset and presented as reads per kilobase of transcript per million mapped reads (RPKM) to compare its transcription level between tumor tissues and adjacent normal tissues in patients. **(B)** Kaplan–Meier plots were used to analyze the effect of Src expression on overall survival and **(C)** disease-free survival. Stratified analysis for the association of Src expression with the survival curve of **(D)** early (I + II) and **(E)** advanced (III + IV) pathological stages of disease is shown. ^∗∗∗^*p* < 0.001 vs. adjacent nomal tissue in TNBC patients.

**FIGURE 7:**
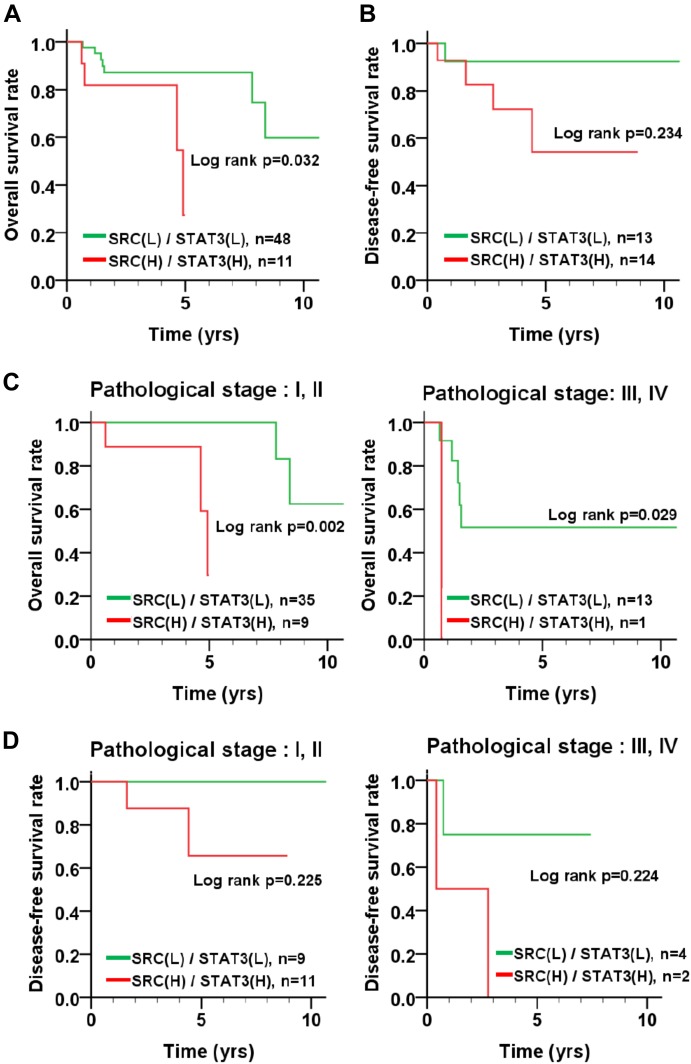
Survival curve of TNBC patients according to the co-expression level of Src and STAT3. **(A)** Kaplan–Meier analyses resulted in an overall survival curve and **(B)** a disease-free survival curve for the level of co-expression of Src and STAT3 in patients with TNBC. The level of co-expression of Src and STAT3 in TNBC patients with early (I + II) and advanced (III + IV) pathological stages of disease was examined for its relation to **(C)** overall survival and **(D)** disease-free survival.

To inspect the relationship between Src and patient survival, we further analyzed Src expression levels in the TNBC dataset obtained from The Cancer Genome Atlas (TCGA). Src gene expression in the tumor tissues of patients with TNBC was significantly higher than that in tumor adjacent normal tissues (Figure 6A; *p* < 0.001, Wilcoxon signed-rank test). Moreover, Kaplan–Meier plots were used to analyze the relationship between Src gene expression and overall survival and disease-free survival in patients with TNBC (Figures 6B,C). Although Src expression level had no significant correlation with either overall or disease-free survival in patients with TNBC (Figures 6B,C), stratification analysis showed that high Src expression was nearly significantly associated with shorter overall survival of patients in the early pathological stages (I + II) of disease (Figures 6D,E). To further examine the association of co-expression of Src and STAT3 with survival in patients, the co-expression level of Src and STAT3 was evaluated using univariate and multivariate Cox proportional hazards models (Table 1). The tumor tissues with high co-expression of Src and STAT3 were significantly associated with a shorter overall survival in both univariate and multivariate analyses (Table 1; crude hazard ratio [CHR] = 3.92, *p* = 0.020; adjusted hazard ratio [AHR] = 3.87, *p* = 0.038), but this co-expression was not statistically significant with disease-free survival (Table 1). Kaplan–Meier analysis of the stratification model indicated that a high co-expression level of Src and STAT3 was associated with poor overall survival in both early (Figure 7; I + II, *p* = 0.002) and advanced (III + IV, *p* = 0.029) pathological stages. Although there was a trend for the association of high co-expression of Src and STAT3 with poor disease-free survival, it was not a statistically significant relationship, likely due to the limited sample size in each group.

**Table 1:** Co-expression of SRC and STAT3 as related to overall survival and disease-free survival in TNBC patients.

Variable	No. (%)	CHR (95% CI)	*p*-value^∗^	AHR (95% CI)	*p*-value^†^
**Overall survival**					
SRC (L) STAT3 (L)	48 (39.0)	1.00		1.00	
Either	64 (52.0)	0.71 (0.28–1.76)	0.453	0.97 (0.35–2.68)	0.949
SRC (H) STAT3 (H)	11 (8.9)	**3.92 (1.24–12.36)**	**0.020**	**3.87 (1.08–13.80)**	**0.039**
**Disease-free survival**					
SRC (L) STAT3 (L)	13 (15.7)	1.00		1.00	
Either	56 (67.5)	0.90 (0.22–3.65)	0.884	1.38 (0.17–11.52)	0.768
SRC (H) STAT3 (H)	14 (16.9)	1.60 (0.33–7.89)	0.561	2.09 (0.19–23.34)	0.549


*Abbreviations: SCC, squamous cell carcinoma; CHR, crude hazard ratio; CI, confidence interval; AHR, adjusted hazard ratio.*

*^∗^*p* values were estimated by Cox’s regression analysis.*

*^†^*p* values were estimated by multivariate Cox’s regression analysis.*

## Discussion

There is no specific biomarker or target for TNBC during chemotherapy thus far, which leads to poor outcomes for patients with TNBC. Kinases are involved in many biological processes that are associated with catabolic homeostasis and diseases. Several kinases have been identified as oncogenes that promote tumorigenesis and resistance of radiation or chemotherapeutic agents. Targeting these oncogenic kinases with either monoclonal antibodies or small molecular inhibitors provides promising antitumor effects. However, little is known about the role of kinases on drug resistance in TNBC cells. Herein, we screened kinome siRNA library for chemoresistant kinase in TNBC cells and report the following findings: first, Src kinase is the most resistant kinase in TNBC cells in response to chemotherapeutic drug doxorubicin. Second, ablation of Src reduced phosphorylation AKT and STAT3 in TNBC cells. Src and STAT3 highly activated TNBC cells were also more resistant to doxorubicin. Third, Src expression was higher in tumor tissues compared to adjacent normal tissues in patients with TNBC. High co-expression of Src and STAT3 was further associated with poor prognosis, suggesting Src-STAT3 axis might be involved in chemoresistance in TNBC cells.

Genome-wide screening with a siRNA library has been employed to identify many novel regulators associated with diseases, such as the polo-like kinase 1 for cancer (Hu et al., 2009), β2 subunit of the coatomer protein complex for virus infection (de Wilde et al., 2015) and the dual-specificity tyrosine-(Y)-phosphorylation regulated kinase 1A for neurodegenerative diseases (Azorsa et al., 2010). Although the screening system provides a powerful tool for biomedical research, it may have been biased by the primary and validation assays. We used the MDA-MB-231 cells in our primary assay and validated the potential genes in both MDA-MB-231 and Hs578T TNBC cells. Several genes appeared to be involved in the chemoresistance of MDA-MB-231 cells, but these genes did not exert any effects in Hs578T TNBC cells, such as the serine/threonine-protein kinase NIM1, C-terminal Src kinase (CSK) and ROS proto-oncogene 1 (ROS1). These kinases may be overexpressed or activated in MDA-MB-231 cells but not in Hs578T cells. ROS1 is a receptor tyrosine kinase that contains an N-terminal extracellular domain and a C-terminal kinase domain (Acquaviva et al., 2009). Gene rearrangement of the 3′ UTR region of ROS1 with many different genes occurs in several cancer cells and activates downstream effectors to promote tumorigenesis, including ERK, AKT, and STS3 (Davies and Doebele, 2013). These results suggest that ROS1 activation may be involved in doxorubicin resistance in MDA-MB-231 cells but not Hs578T cells. Further study is required to examine the differential roles of ROS1 in these TNBC cells. Moreover, dasatinib suppresses chemoresistance in chondrosarcoma cells, particularly in cells with TP53 mutations (van Oosterwijk et al., 2013). Our current study also showed that Src-silenced breast cancer cells with a TP53 mutation, such as MDA-MB-231, Hs578T, and T47D cells, were relatively more sensitive to doxorubicin than that in MCF-7 cells, which have wild-type TP53. More importantly, most of patients with TNBC have TP53 mutation (∼75%) (Liu et al., 2018). Though TP53 mutation may be involved in the resistant mechanisms for DNA damaged drugs, several mechanisms might also confer to chemoresistance in TNBC cells. Thus, investigating chemoresistant targets in TNBC cells with TP53 mutation would be able to reflect the situation in clinical settings. However, the role of TP53 in Src-modulated chemoresistance in breast cancer cells requires more work to be verified.

Src tyrosine kinases are overexpressed in TNBC and promote receptor tyrosine kinase phosphorylation, which affects cell adhesion and migration (Hochgrafe et al., 2010). The Src protein level is higher in tumor tissues of patients with TNBC than in patients with non-TNBC (Tryfonopoulos et al., 2011). Combination treatment with dasatinib and cisplatin has synergistic effects on cancer suppression in TNBC cells (Tryfonopoulos et al., 2011), supporting our notion that Src might be involved in the chemoresistance of TNBC cells. Nevertheless, the results of TCGA data analysis showed that Src mRNA level is not statistically correlated with overall and disease-free survival in patients with TNBC. The phosphorylation of Src at Tyr416, which activates Src, is associated with poor disease-free survival in patients with colorectal cancer and cervical cancer (Hou et al., 2013; Kopetz et al., 2014). These results raise a possibility that Src activation may be a more precise way to determine its correlation with disease in TNBC patients, but further study is required to verify this idea.

Regarding the mechanisms of Src-enhanced chemoresistance, Src confers to AKT/MTOR activation in acute myeloid leukemia for cancer stemness (Li et al., 2014), which is highly associated with drug resistance (Prieto-Vila et al., 2017). Silencing Src reduces expression of Aldehyde dehydrogenase isoform 1 (ALDH1), a cancer stemness marker, and sphere formation in lung cancer cells (Lee et al., 2018). Besides, Src can phosphorylate caspase-8 at tyrosin 397 to suppress apoptosis (Tsang et al., 2016). Src may also inhibit apoptosis via directly phosphorylation at tyrosin 530 of Ku-70, which is a crucial protein to repair DNA double-strand breaks (Morii et al., 2017). These results suggest Src could activate several pathways to cause chemoresistance in cancer cells.

Src can increase pro-inflammatory cytokines production for tumor development and activate survival effectors for chemoresistance (Su et al., 2014; Tsai et al., 2017), including TNF-α, IL-1β, IL6, phosphoinositide 3-kinases (PI3Ks), AKT and STAT3. Dasatinib has been shown to inhibit the growth of TNBC cell lines *in vitro* when used in combination with standard chemotherapy, such as cisplatin, or when used as a single agent (Finn et al., 2007). The PI3K/AKT/mTOR pathway is frequently deregulated in TNBC and inhibitors against this pathway are being developed in preclinical or early phase clinical trials (Massihnia et al., 2016). Rapamycin and its analogs temsirolimus, everolimus, and deforolimus, inhibitors of mTOR, are undergoing clinical evaluation for treatment of a variety of cancers, including breast cancer (Minami et al., 2011), supporting our notion that Src-AKT-STAT3 signaling may be crucial for chemoresistance in TNBC cells. Besides Src-STAT3 in chemoresistance of TNBC, several mechanisms might be involved in the chemoresistance of TNBC as reported previously, including (i) ABC transporters efflux, (ii) NFκB pathway, (iii) increase of anti-apoptosis and detoxification proteins (O’Reilly et al., 2015) and (iv) gene amplification of proto-oncogenes, such as MYC and JAK2 (Wein and Loi, 2017). However, the crosstalk among the pathways and effects of the other pathways on Src-mediated chemoresistance in cell culture model and clinical relevance would need further studies to verify.

In addition, STAT3 has been widely recognized as an oncogene in various cancers and has been confirmed to be constitutively active in TNBC (Deng et al., 2012; Xiong et al., 2014). Knockdown of STAT3 enhanced cisplatin-induced apoptosis in ovarian cancer and non-small cell lung cancer cells (Yin et al., 2012; Han et al., 2013). Although dasatinib is more effective for cancer cells with aberrant Src activation (Zhang and Yu, 2012), no Src inhibitors successfully suppresses tumors in cancer patients when used alone, possibly because there is no suitable biomarker to monitor Src activation in tumor tissues or because the treatment triggers alternative routes for resistance to Src inhibition (Zhang and Yu, 2012). Our results suggest that the Src-STAT3 axis might play an important role in the chemoresistance of TNBC cells and may be used to evaluate if tumors can be treated with Src inhibitors during chemotherapy.

## Author Contributions

Y-DT, P-FL, and J-YL performed most experiments, and L-FL (Figures 5A,B), S-YK (Figure 4E), C-WH and C-HW (Figures 5C,D) performed the remaining experiments. TCGA data was analyzed by C-HL, H-TC, and MH. C-WS prepared the figures, interpreted data, and wrote the manuscript.

## Conflict of Interest Statement

The authors declare that the research was conducted in the absence of any commercial or financial relationships that could be construed as a potential conflict of interest.

## Footnotes

1 https://xenabrowser.net/heatmap/
